# Natural Remission of Major Periprosthetic Osteolysis following Total Hip Arthroplasty with Metal-on-Metal Bearings

**DOI:** 10.1155/2017/2576196

**Published:** 2017-10-04

**Authors:** Tatsuya Tamaki, Kazuhiro Oinuma

**Affiliations:** Joint Reconstruction Center, Funabashi Orthopedic Hospital, Funabashi, Japan

## Abstract

The natural course of adverse events following the use of metal-on-metal (MoM) bearings in total hip arthroplasty (THA) is not well known. In this article, we report the case of a patient with asymptomatic major acetabular osteolysis following MoM THA that diminished gradually without any surgical intervention. A 58-year-old male underwent one-stage bilateral MoM THA for bilateral osteoarthritis. Four years after THA, major acetabular osteolysis developed in his right hip without any local or systemic symptoms. The patient underwent a careful radiographic and clinical observation without any surgical intervention because he did not want to undergo revision surgery. The lesion gradually diminished after 7 years, and most of the osteolytic area was replaced by newly formed bone at 10 years. He continues to be followed with no evidence of cup loosening or migration. Our observation suggests that a periprosthetic osteolytic change related to the use of MoM bearings has the potential for natural remission.

## 1. Introduction

Concerns regarding the use of metal-on-metal (MoM) bearings in total hip arthroplasty (THA) include elevated metal ion levels in the serum, adverse reactions to metal debris, and subsequent soft tissue and bone destruction [1–4]. Although published Kaplan-Meier survivorship analyses of THA using MoM bearings have been reported to be excellent at the long-term follow-up [5–8], the natural course of adverse reactions related to MoM bearings is currently unavailable from those reports.

From March 2006 to September 2011, 104 consecutive THAs were performed using a modular acetabular MoM component with either a 28 or 32 mm femoral head at our institution. In this series (median follow-up of 8.0 years), pseudotumour formation was clinically observed in one hip (1.0%), and revision surgery was performed for that hip. Follow-up radiographs revealed asymptomatic major acetabular osteolysis in one hip (1.0%), which developed 4–6 years after THA without any symptoms; however, it gradually diminished without surgical intervention after 7 years, and most of the osteolytic area had been replaced by newly formed bone at the final follow-up. To our knowledge, there have been no reports describing the remission of a major osteolytic change related to the use of MoM bearings.

In this article, we report the case of a patient with asymptomatic major acetabular osteolysis following MoM THA that gradually diminished without any surgical intervention.

## 2. Case History

**Figure 1:**
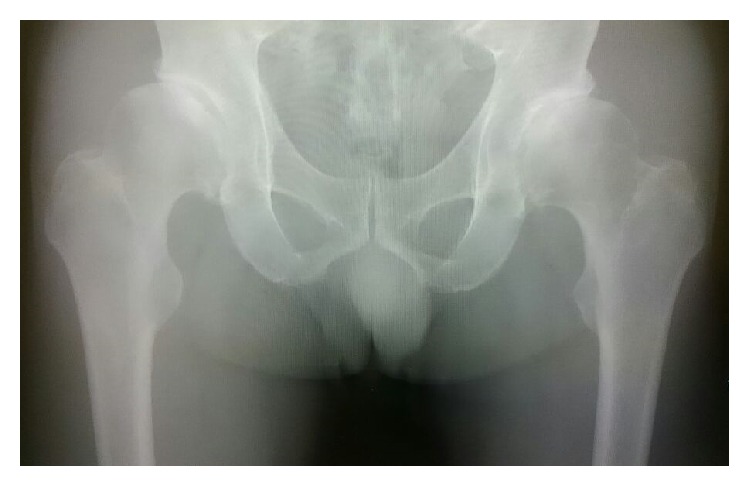
Preoperative anteroposterior X-ray showing bilateral osteoarthritis of the hip.

**Figure 2:**
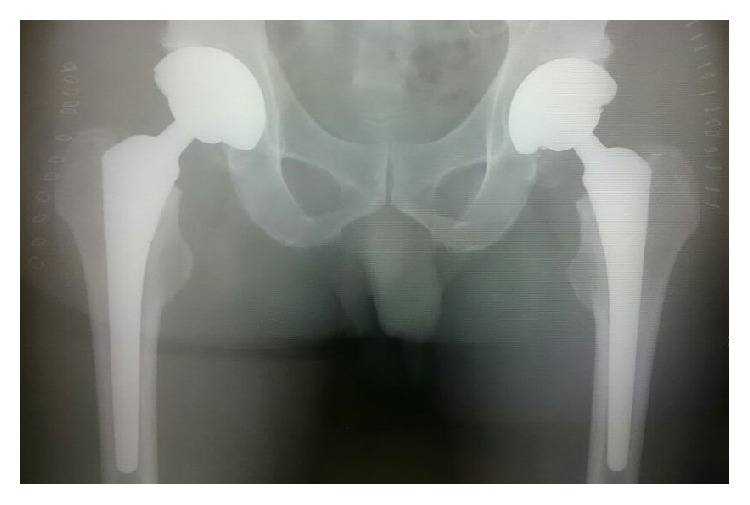
Postoperative anteroposterior X-ray.

A 58-year-old male visited our outpatient clinic complaining of bilateral hip pain over the previous few years. X-rays showed progressive arthritis of the bilateral hip joint (Figure 1). He was engaged in light labour. His height, weight, and body mass index were 173 cm, 72 kg, and 24.1 kg/m^2^, respectively. He had undergone one-stage bilateral THA with MoM bearings in 2006 (Figure 2). The direct anterior approach was used with the patient in the supine position on a standard surgical table. The implant used in the patient was M2a Taper (Biomet, Warsaw, IN) with BiMetric stem (Biomet). The cup diameter was 56 mm and the head diameter and length were 32 mm and +3 mm, respectively. The perioperative course was uneventful, and full weight bearing was allowed immediately after surgery. He was discharged to his own home with the ability to walk with a cane on the 10th day after surgery.

**Figure 3:**
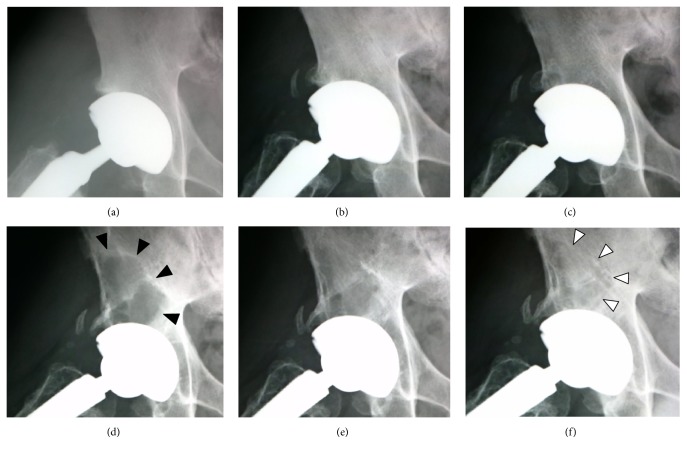
Lauenstein view of the right hip immediately after surgery (a) and at 2 (b), 4 (c), 6 (d), 8 (e), and 10 (f) years after surgery. After 4 years, acetabular osteolysis developed in zones 1 and 2 (black arrow heads), and the lesion was most significant at 6 years after surgery. However, most of the osteolytic area had been replaced by newly formed bone at 10 years (white arrow heads) without evidence of implant loosening.

The degree of acetabular anteversion and abduction measured using the method described by Dorr and Wan [9] were 21 degrees and 39 degrees for the right side and 18 degrees and 37 degrees for the left side, respectively. Both cups were placed within Lewinnek et al.'s [10] safe zone. Four years after THA, major acetabular osteolysis developed in his right hip in Delee and Charnley [11] zones 1 and 2 (Figure 3(c)), whereas no osteolytic change was observed in the left side. The osteolytic change was most significant at 6 years after surgery (Figure 3(d)). He did not complain of hip pain or discomfort throughout the follow-up. No local symptoms, including swelling, tenderness, or warmth, were evident from a physical examination. The patient underwent a careful physical and radiographic observation because he had no symptoms and he did not want to undergo revision surgery. The lesion gradually diminished after 7 years. Finally, most of the osteolytic area had been replaced by newly formed bone at 10 years (Figure 3(f)). No evidence of cup loosening or migration was observed throughout the follow-up period.

## 3. Discussion

There are many factors still unknown about the adverse events related to the MoM bearings in THA because there have been few longitudinal studies of these events. Therefore, indications for revision have not yet been established. Some authors have reported a natural history of asymptomatic pseudotumours [12, 13] following the use of MoM bearings and noted that pseudotumours have the potential for natural remission in some patients. However, to our knowledge, there is no report describing the remission of a major osteolytic change related to the use of MoM bearings without surgical intervention.

We had a patient who demonstrated natural remission of major periprosthetic osteolytic changes after THA using a 32 mm diameter MoM bearing. It remains unclear why osteolysis appeared only in the right hip. However, we speculated that asymmetry in local metal ion levels were responsible for the development of osteolysis in the right hip. Because we had an only single case, we cannot generalise our result. Further, we cannot compare our results with large-diameter MoM bearings because reported metal ion levels after small- and large-diameter MoM THA are different [14, 15]. Although adverse reactions to metal debris can progressively erode tissues in certain cases and revision surgery is the radical solution in such situations, it is worth remembering that periprosthetic osteolytic changes related to the use of MoM bearings have the potential for natural remission without surgical intervention. Careful observation could be an alternative for patients with asymptomatic periprosthetic osteolytic changes following MoM THA.

There were some limitations in this case report. First, the serum metal ion level was not evaluated. A one-stage bilateral procedure could contribute to the elevation of the serum metal ion level compared to a unilateral procedure. Second, computed tomography or magnetic resonance image were not undertaken. Therefore, subclinical reactions such as a small pseudotumour or fluid collection may have been overlooked. Moreover, we could not deny the possibility that there was still significant osteolysis behind the cup that was not evident on the plain X-ray views.

Our observation suggests that a periprosthetic osteolytic change related to the use of MoM bearings has the potential for natural remission.

## Additional Points

*Summary*. We reported the case of a patient with asymptomatic major acetabular osteolysis following MoM THA that gradually diminished without surgical intervention. Careful observation could be an option for patients with asymptomatic osteolysis following MoM THA.
